# Is There an Added Value of Dual-Time-Point [^68^Ga]Ga–Fibroblast Activation Protein Inhibitor (FAPI) PET/CT in Differentiating Malignant and Benign Uptake Findings?

**DOI:** 10.3390/cancers18121963

**Published:** 2026-06-17

**Authors:** Akram Al-Ibraheem, Serin Moghrabi, Baraa Alsyouf, Marwah Abdulrahman, Mahd Al-Foqaha, Farah Al-Tameemi, Bara’ah Bashabsheh, Saad Ruzzeh, Dimah Khalid Jiad, Ahmed Firas Al-Hammouri, Hongcheng Shi, Ahmed Saad Abdlkadir, Asem Mansour

**Affiliations:** 1 Department of Nuclear Medicine, King Hussein Cancer Center (KHCC), Amman 11942, Jordan; 2 Division of Nuclear Medicine, Department of Radiology and Nuclear Medicine, University of Jordan, Amman 11942, Jordan; 3 Department of Obstetrics and Gynecology, Jordan Hospital, Amman 11152, Jordan; 4 Internal Medicine Department, Royal Medical Services, Amman 11855, Jordan; 5 Department of Nuclear Medicine, Zhongshan Hospital, Fudan University, Shanghai 200032, China; 6 Department of Nuclear Medicine, Radiotherapy and Nuclear Medicine Hospital, Bab Al-Muadham, Baghdad 10047, Iraq; 7 Department of Radiology, King Hussein Cancer Center (KHCC), Amman 11942, Jordan

**Keywords:** [^68^Ga]Ga-FAPI PET/CT, dual-time-point imaging, SUVmax, TBRmax, MTV, TLU

## Abstract

**Background**: [^68^Ga]Ga-FAPI PET/CT demonstrates high sensitivity for tumor detection but limited specificity because benign fibro-inflammatory conditions may also show tracer uptake. Dual-time-point imaging has been proposed to improve lesion characterization by assessing temporal changes in uptake. This study evaluated whether delayed imaging provides incremental diagnostic value over standard early imaging. **Methods**: This retrospective lesion-based study evaluated dual-time-point [^68^Ga]Ga-FAPI PET/CT imaging. SUVmax, maximal tumor-to-background ratio (TBRmax), metabolic tumor volume (MTV), and total lesion uptake (TLU) were measured on early and delayed scans. Diagnostic performance for differentiating malignant from benign uptake findings was assessed using receiver operating characteristic analysis. **Results**: A total of 123 patients underwent dual-time-point imaging at approximately 26 and 65 min post-injection. Overall, 620 [^68^Ga]Ga-FAPI uptake findings were analyzed, including 307 malignant uptake findings and 313 benign findings. SUVmax decreased significantly over time in both malignant and benign uptake findings, with a greater decline in benign findings (%ΔSUVmax −7.7% vs. −3.6%, *p* = 0.0045). TBRmax increased modestly in malignant uptake findings, while MTV and TLU remained largely stable. SUVmax and TBRmax were significantly higher in malignant uptake at both imaging time points; however, diagnostic performance remained moderate (AUCs of 0.65–0.69) due to substantial overlap between lesion types. Delayed imaging did not improve diagnostic accuracy compared with early imaging, and delta parameters demonstrated poor performance (AUC ~0.5). Similar findings were observed across tumor subgroups. **Conclusions**: In this lesion-based retrospective cohort, delayed dual-time-point [^68^Ga]Ga-FAPI PET/CT did not demonstrate meaningful incremental diagnostic value over early imaging. A standard early acquisition appears adequate for routine practice, although lesion interpretation remains dependent on clinical, morphologic, and, when needed, histopathologic correlation.

## Simple Summary

Dual-time-point [^68^Ga]Ga-FAPI PET/CT imaging has been proposed as a way to improve the distinction between cancerous and non-cancerous uptake by evaluating how tracer uptake changes over time. However, it remains unclear whether delayed imaging provides meaningful additional diagnostic benefit compared with standard early imaging. In this study, we evaluated early and delayed [^68^Ga]Ga-FAPI PET/CT scans in patients with a wide range of cancers and compared their ability to differentiate malignant from benign findings. Although some changes in tracer uptake were observed over time, delayed imaging did not significantly improve diagnostic accuracy. Early imaging alone provided comparable performance while reducing scan time and simplifying workflow. These findings suggest that a single early acquisition may be sufficient for routine clinical practice, which could improve patient convenience and imaging department efficiency while maintaining diagnostic reliability.

## 1. Introduction

Fibroblast activation protein (FAP) is a membrane-bound serine protease highly expressed in activated fibroblasts, particularly cancer-associated fibroblasts (CAFs), where it plays a central role in extracellular matrix remodeling, tumor progression, and stromal signaling [1,2]. In contrast, FAP expression in most normal adult tissues is minimal, making it an attractive target for molecular imaging [3]. Radiolabeled fibroblast activation protein inhibitors (FAPIs), particularly [^68^Ga]Ga-labeled compounds such as FAPI-04 and FAPI-46, have demonstrated rapid tumor uptake, high tumor-to-background contrast, and favorable pharmacokinetics across a wide range of malignancies [4].

In oncology, [^68^Ga]Ga-FAPI PET/CT has demonstrated high uptake across a wide range of malignancies, including pancreatic, breast, colorectal, and lung cancers [5,6,7]. By targeting cancer-associated fibroblasts within the tumor stroma, it provides a complementary perspective to tumor-cell-based imaging and is particularly effective in tumors with prominent desmoplastic reaction [7,8,9]. Clinically, [^68^Ga]Ga-FAPI PET/CT has shown value in tumor detection, staging, and delineation, often with improved lesion conspicuity due to low background activity in normal tissues. These characteristics have positioned [^68^Ga]Ga-FAPI PET/CT as a promising alternative or complement to [^18^F]F-FDG PET/CT in oncologic imaging [10,11,12].

Also, beyond oncology, increasing evidence has shown that [^68^Ga]Ga-FAPI uptake is not tumor-specific but reflects fibroblast activation in a broad spectrum of nonmalignant conditions, including inflammation, fibrosis, and tissue remodeling [13,14,15]. This overlap between malignant and benign fibroblast activation represents a key diagnostic challenge and underscores the need for improved interpretation strategies [16,17].

One emerging approach to improve diagnostic specificity is dual-time-point [^68^Ga]Ga-FAPI hybrid imaging, which exploits the dynamic behavior of tracer uptake over time. [^68^Ga]Ga-FAPI tracers demonstrate rapid tumor accumulation and fast background clearance, enabling early imaging (typically ~10–30 min post-injection) with high lesion detectability, while delayed imaging (~50–90 min or later) may further enhance contrast due to continued tumor retention and washout from non-target tissues [18,19,20]. Preliminary dual-time-point studies indicate that [^68^Ga]Ga-FAPI uptake may change over time in a lesion-dependent manner, but reported kinetic patterns are heterogeneous, and the optimal imaging time point has not yet been established [18,19,20]. Current clinical practice largely relies on a single early acquisition, and the added value of delayed or dual-time-point imaging, particularly in differentiating malignant from benign uptake findings and improving diagnostic performance, has yet to be clearly established [21,22].

Given the increasing clinical use of [^68^Ga]Ga-FAPI PET/CT and the recognized overlap between malignant and benign fibroblast activation, it is important to determine whether dual-time-point imaging provides meaningful incremental diagnostic value beyond single-time-point imaging. This study aims to evaluate dual-time-point [^68^Ga]Ga-FAPI PET/CT by comparing early and delayed acquisitions in terms of lesion detectability, quantitative metrics, and diagnostic performance for both malignant and benign uptake findings, to determine whether early imaging, delayed imaging, or their combination provides the most accurate and clinically useful assessment.

## 2. Materials and Methods

### 2.1. Study Design and Patient Population

This retrospective single-center study was conducted at King Hussein Cancer Center (KHCC), Amman, Jordan, and included patients who underwent [^68^Ga]Ga-FAPI PET/CT imaging between September 2022 and September 2025. A total of 141 patients were screened. Of these, 15 patients who underwent single-time-point imaging were excluded. The final study cohort comprised 123 patients who underwent dual-time-point imaging with both early and delayed acquisitions. Inclusion criteria required the availability of both early and delayed whole-body [^68^Ga]Ga-FAPI PET/CT scans.

Patients included both therapy-naive and previously treated individuals, as dual-time-point imaging was performed for various clinical indications, including staging, follow-up, and treatment response assessment.

The study protocol was approved by the institutional review board (IRB No. 25 KHCC 316). Given the retrospective nature of the study, the requirement for informed consent was waived. All procedures were performed in accordance with institutional guidelines and the principles of the Declaration of Helsinki.

### 2.2. Radiotracer Preparation and Imaging Protocol

All patients received an intravenous injection of [^68^Ga]Ga-FAPI-04 at a weight-based administered activity (MBq/kg), approximately 1.8–2.6 MBq/kg. PET/CT imaging was performed using a United Imaging uMI 780 PET/CT (Shanghai, China).

Dual-time-point imaging was performed for all patients. The early acquisition was obtained at approximately 20 ± 10 min post-injection, followed by a delayed acquisition at approximately 50 ± 10 min post-injection. For each time point, whole-body PET/CT imaging was acquired from the skull base to mid-thigh using 5–6 bed positions (2 min per bed position). Low-dose CT was performed for attenuation correction and anatomical localization. PET images were reconstructed using an ordered-subset expectation maximization (OSEM) iterative algorithm, incorporating corrections for attenuation, scatter, randoms, and decay, with a slice thickness of 3 mm. Reconstruction parameters were kept consistent across time points for each patient. After reconstruction, post-processing and image fusion were performed using Syngo.via software (version VB40, Siemens Healthineers, Erlangen, Germany) for image analysis.

### 2.3. Image Interpretation and Lesion Selection

Image analysis was performed independently by four senior nuclear medicine physicians in training with dedicated experience in PET/CT interpretation and quantitative image analysis. To ensure consistency and reduce interobserver variability, all interpretations were subsequently reviewed and validated by two senior board-certified nuclear medicine consultants, each with more than 10 years of experience in PET/CT imaging and interpretation.

A lesion-based analysis was adopted, with uptake evaluated across all organ systems for each patient. To standardize analysis and minimize overrepresentation of patients with high lesion burden, only the most avid lesion per involved organ system was included. Both suspected malignant and benign sites of uptake were analyzed.

### 2.4. Quantitative PET Analysis

Semi-quantitative analysis was performed using dedicated workstation software (syngo.via VB40, Siemens Healthineers, Erlangen, Germany). For each selected lesion, measurements were obtained on both early and delayed PET images. The maximum standardized uptake value (SUVmax) was determined by placing a region of interest (ROI) over the lesion.

In addition, volumetric parameters, including metabolic tumor volume (MTV) and total lesion uptake (TLU), were calculated using a semi-automated threshold-based segmentation method, with the threshold set at 40% of the lesion SUVmax [23]. TLU was calculated as the product of MTV and SUVmean (TLU = MTV × SUVmean), analogous to total lesion glycolysis (TLG) used in [^18^F]F-FDG PET imaging and consistent with previously published FAPI PET studies [24,25]. The same segmentation approach was applied consistently across early and delayed time points.

Background activity was assessed using a region of interest placed in the blood pool, typically at the level of the aortic arch. The maximal tumor-to-background ratio (TBRmax) was calculated by normalizing lesion SUVmax to blood pool activity.

To evaluate temporal changes in tracer uptake, differences between early and delayed imaging were calculated for each quantitative parameter, including %ΔSUVmax, %ΔMTV, and %ΔTLU.

### 2.5. Lesion Classification and Reference Standard

Sites of uptake were categorized as malignant or benign. Histopathological confirmation was used as the reference standard when available. In a tertiary care setting, histopathology was obtained for a subset of uptake findings, primarily for primary tumors and selected equivocal findings. The remaining uptake findings were classified using a composite reference standard by incorporating imaging follow-up, clinical data, and multidisciplinary expert consensus. Consensus interpretation was performed by experienced nuclear medicine physicians, taking into account lesion morphology, anatomical distribution, concordance with known disease patterns, temporal behavior on follow-up imaging, relevant clinical history, and additional diagnostic information. A minimum follow-up period of 3–6 months, including serial imaging (PET/CT, contrast-enhanced CT, or MRI) and clinical assessment, was required when histopathological confirmation was not available. The distribution of reference standards is summarized in Appendix A, Table A1.

Uptake findings were considered benign if they demonstrated imaging features consistent with non-malignant etiologies, remained stable in size and tracer uptake, or showed resolution on subsequent imaging, with no clinical evidence of malignancy during follow-up. Conversely, uptake findings were classified as malignant if they demonstrated progression on follow-up imaging, were associated with clinical or biochemical evidence of active disease, or were consistent with known metastatic patterns.

### 2.6. Study Endpoints

The primary objective of this study was to evaluate whether delayed dual-time-point [^68^Ga]Ga-FAPI PET/CT imaging provides incremental diagnostic value over early imaging in characterizing malignant and benign uptake findings.

Secondary objectives included a comprehensive comparison of quantitative uptake parameters, namely, SUVmax, TBRmax, MTV, and TLU, between early and delayed imaging in both malignant and benign uptake findings. In addition, temporal changes in these parameters were assessed using both absolute (Δ) and relative (%Δ) differences to characterize tracer kinetics over time.

Further analyses included comparison of quantitative uptake between malignant and benign uptake findings at each imaging time point, and assessment of the diagnostic performance of early, delayed, and delta parameters for lesion characterization.

### 2.7. Statistical Analysis

Statistical analysis was performed using Python (version 3.14.3; Python Software Foundation, Wilmington, DE, USA). Descriptive demographic variables are presented as mean ± SD, while imaging parameters are reported as median [IQR] due to non-normal distribution. Normality was assessed using the Shapiro–Wilk test. Quantitative variables demonstrated non-normal distribution (*p* < 0.05) and are therefore presented as median, with non-parametric tests applied.

Paired comparisons between early and delayed imaging parameters (SUVmax, TBRmax, MTV, and TLU) were performed using the Wilcoxon signed-rank test. Differences in quantitative parameters between malignant and benign uptake findings were evaluated using the Mann–Whitney U test.

Temporal changes in quantitative parameters were assessed using both absolute differences (Δ) and relative percentage changes (%Δ), calculated as [(delayed − early)/early] × 100. Comparisons of Δ and %Δ between malignant and benign uptake findings were performed using the Mann–Whitney U test.

Receiver operating characteristic (ROC) curve analysis was performed to evaluate the diagnostic performance of quantitative parameters at each time point and their temporal changes in differentiating malignant from benign uptake findings. The area under the curve (AUC), optimal cutoff values (determined using the Youden index), sensitivity, specificity, positive predictive value (PPV), and negative predictive value (NPV) were calculated.

All analyses were performed at the lesion level. A two-sided *p*-value of < 0.05 was considered statistically significant. As multiple uptake findings could originate from the same patient, statistical independence between observations cannot be fully assumed. Therefore, the results should be interpreted as exploratory, reflecting lesion-based trends rather than strictly independent measurements.

## 3. Results

### 3.1. Baseline Patient and Lesion Characteristics

A total of 123 patients were included in this study. The mean age was 54.4 ± 15.9 years (range, 16–87 years). The cohort comprised 56 male and 67 female patients. Dual-time-point imaging was performed, with a mean first acquisition time of 26.0 ± 8.5 min and a mean second acquisition time of 65.1 ± 11.9 min following tracer administration. The mean interval between the two acquisitions was 39.2 ± 8.3 min.

The cohort represented a heterogeneous oncologic population. The most common primary diagnoses were colorectal cancer (26.0%) and pancreatic cancer (18.7%), followed by gastric, appendiceal, and thyroid cancers (each 9.8%). Additional tumor types included cholangiocarcinoma, ovarian cancer, and carcinoma of unknown origin. Less frequent malignancies were grouped as “others,” and four patients had dual primary malignancies.

Detailed tumor staging and histological grading data were not consistently available for all patients and were therefore not included in the analysis.

The primary indications for [^68^Ga]Ga-FAPI PET/CT imaging were follow-up (41.9%), therapy response assessment (21.6%), and staging (20.4%), followed by restaging, suspected recurrence, and post-surgical evaluation. Benign uptake findings were not primary indications for imaging and were incidentally identified during whole-body PET/CT performed for oncologic purposes.

The most frequent treatment combinations were chemotherapy and surgery (33/123, 26.8%), followed by surgery alone (26/123, 21.1%) and chemotherapy alone (14/123, 11.4%). Triple-modality therapy (chemotherapy, radiotherapy, and surgery) was observed in 10 patients (8.1%), while other combinations were less common. Notably, 34 patients (27.6%) had not received prior therapy.

A summary of baseline patient characteristics is presented in Table 1.

A total of 620 uptake findings were analyzed, including 307 malignant uptake findings (49.5%) and 313 benign uptake findings (50.5%). Not all uptake findings were histopathologically confirmed; a proportion were classified based on the composite reference standard as described in Section 2. The distribution of reference standards is presented in Appendix A, Table A1.

### 3.2. Temporal Changes in Quantitative Parameters Between Early and Delayed [^68^Ga]Ga-FAPI PET/CT

#### 3.2.1. Malignant Uptake Findings

In malignant uptake findings, SUVmax demonstrated a modest but statistically significant decrease on delayed imaging compared with early imaging (7.90 [4.80–12.60] vs. 7.50 [4.30–12.60]; Δ −0.30 [−1.40–0.90]; *p* = 0.0194). In contrast, TLU showed a slight reduction between early and delayed scans (17.20 [7.30–74.94] vs. 18.40 [7.40–65.29]; Δ −0.29 [−4.19–3.55]), which was not statistically significant (*p* = 0.4262). TBRmax demonstrated a small but statistically significant increase over time (4.30 [2.63–7.12] vs. 5.00 [2.71–7.83]; Δ 0.18 [−0.42–0.98]; *p* = 0.0001). No significant change was observed for MTV (4.00 [1.89–15.20] vs. 4.60 [1.93–15.40]; Δ 0.00 [−0.96–1.40]; *p* = 0.4462), indicating relative stability of lesion volume.

#### 3.2.2. Benign Findings

In benign findings, SUVmax similarly demonstrated a significant decrease on delayed imaging (4.70 [3.16–7.70] vs. 4.20 [2.60–7.40]; Δ −0.30 [−1.28–0.20]; *p* < 0.0001). TLU showed a decrease over time (25.40 [9.61–76.50] vs. 21.90 [9.28–73.20]; Δ −1.50 [−11.40–4.25]), which was statistically significant (*p* = 0.0012). MTV demonstrated a mild increase between early and delayed imaging (8.91 [4.40–20.01] vs. 10.15 [4.79–20.85]; Δ 0.23 [−1.92–3.53]; *p* = 0.0491). TBRmax showed minimal change over time (2.79 [1.77–4.70] vs. 2.86 [1.88–4.59]; Δ 0.10 [−0.39–0.51]; *p* = 0.0517), without statistical significance (Table 2).

Overall, both malignant uptake findings and benign findings demonstrated a significant reduction in SUVmax on delayed imaging. TBRmax showed a modest increase over time, reaching statistical significance only in malignant uptake findings, while MTV and TLU remained largely unchanged in both groups. These findings indicate that although measurable temporal changes in [^68^Ga]Ga-FAPI uptake occur between early and delayed imaging, these changes are limited and inconsistent across quantitative parameters.

### 3.3. Percentage Change Analysis

Comparison of percentage changes (%Δ) between malignant and benign findings revealed a statistically significant difference for SUVmax, with malignant uptake findings demonstrating a smaller reduction compared to benign findings (−3.6 [−17.4–11.3] vs. −7.7 [−24.2–4.6]; *p* = 0.0045).

In contrast, no significant differences were observed between malignant and benign uptake findings for TBRmax (5.1 [−10.0–22.8] vs. 3.6 [−12.6–23.7]; *p* = 0.4079), MTV (0.0 [−22.2–36.4] vs. 4.9 [−22.2–43.7]; *p* = 0.3616), or TLU (−2.3 [−23.4–20.5] vs. −7.0 [−33.1–29.2]; *p* = 0.1393) (Table 3).

These findings suggest that relative temporal changes in [^68^Ga]Ga-FAPI uptake are largely comparable between malignant and benign uptake findings across most quantitative parameters, with the exception of SUVmax, which demonstrates a modest but statistically significant difference.

### 3.4. Comparison of Quantitative Parameters Between Malignant and Benign Uptake Findings

Comparative analysis of quantitative parameters demonstrated significant differences between malignant and benign uptake findings at both early and delayed time points (Table 4).

SUVmax was significantly higher in malignant uptake findings compared to benign uptake findings at both early (7.9 [4.8–12.6] vs. 4.7 [3.2–7.7]; *p* < 0.0001) and delayed imaging (7.5 [4.3–12.6] vs. 4.2 [2.6–7.4]; *p* < 0.0001). Similarly, TBRmax was significantly greater in malignant uptake findings at both early (4.3 [2.6–7.1] vs. 2.8 [1.8–4.7]; *p* < 0.0001) and delayed time points (5.0 [2.7–7.8] vs. 2.9 [1.9–4.6]; *p* < 0.0001).

In contrast, MTV was significantly higher in benign uptake findings compared to malignant uptake findings at both early (8.9 [4.4–20.0] vs. 4.0 [1.9–15.2]; *p* < 0.0001) and delayed imaging (10.1 [4.8–20.9] vs. 4.6 [1.9–15.4]; *p* < 0.0001). For TLU, benign uptake findings demonstrated higher values at the early time point (25.4 [9.6–76.5] vs. 17.2 [7.3–74.9]; *p* = 0.0465). However, this difference was not statistically significant on delayed imaging (21.9 [9.3–73.2] vs. 18.4 [7.4–65.3]; *p* = 0.1191).

### 3.5. Diagnostic Performance of Early, Delayed, and Delta Parameters

**Figure 1:**
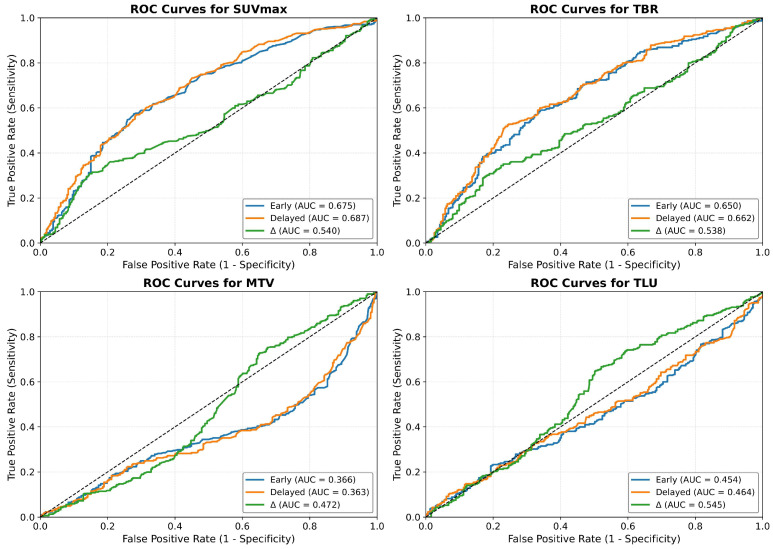
Receiver Operating Characteristic Curves for SUVmax, TBRmax, TLU, and MTV. The dashed diagonal line indicates the reference line for random classification (AUC = 0.5).

Receiver operating characteristic (ROC) analysis was performed to evaluate the ability of early, delayed, and delta quantitative parameters to differentiate malignant from benign uptake findings (Figure 1 and Table 5).

Among all evaluated parameters, SUVmax demonstrated the highest diagnostic performance, with comparable area under the curve (AUC) values for early and delayed imaging (0.674 [95% CI: 0.631–0.717] vs. 0.687 [95% CI: 0.643–0.729], respectively). The optimal thresholds yielded moderate sensitivity and specificity (early: 57.3% and 72.2%; delayed: 60.3% and 68.4%). In contrast, ΔSUVmax demonstrated limited discriminative ability (AUC 0.566), with high sensitivity (72.0%) but low specificity (39.3%), indicating poor overall diagnostic performance.

Similarly, TBRmax showed moderate discriminative ability, with slightly higher AUC on delayed imaging compared to early imaging (0.662 vs. 0.649). The delayed TBRmax threshold provided higher specificity (75.4%) but lower sensitivity (52.8%), while early TBRmax demonstrated a more balanced performance (sensitivity 59.0%, specificity 65.8%). However, ΔTBRmax showed poor diagnostic utility (AUC 0.520), characterized by very high sensitivity (90.5%) but extremely low specificity (17.3%).

In contrast, MTV demonstrated poor diagnostic performance across all time points, with AUC values below 0.40 for both early (0.365) and delayed imaging (0.362). Although specificity was very high (>98%), sensitivity was extremely low (<3%), limiting its clinical utility. Similarly, ΔMTV showed no meaningful discriminative ability (AUC 0.480).

TLU also demonstrated limited diagnostic value, with low AUC values for early (0.454) and delayed imaging (0.464). While delayed TLU showed high specificity (92.9%), sensitivity remained low (10.5%). ΔTLU showed only marginal improvement (AUC 0.535) but remained inadequate for reliable lesion characterization.

Overall, delayed imaging did not provide a meaningful improvement in diagnostic performance compared with early imaging. SUVmax and TBRmax demonstrated moderate discriminative ability at both time points, with comparable AUC values, while MTV and TLU showed poor diagnostic performance. Furthermore, delta parameters did not improve lesion characterization, with AUC values approaching 0.5. Formal comparison of the early and delayed SUVmax ROC curves using DeLong’s test demonstrated no statistically significant difference between the two AUCs (0.674 vs. 0.687; *p* = 0.18).

Collectively, these findings indicate that early [^68^Ga]Ga-FAPI PET/CT imaging provides diagnostic performance comparable with delayed imaging, with no statistically significant improvement achieved through dual-time-point acquisition.

### 3.6. Subgroup Analysis by Cancer Type

Subgroup analysis was performed for cancer types with a minimum of 10 patients to ensure adequate statistical representation. The diagnostic performance of SUVmax was evaluated by comparing malignant uptake findings within each cancer type against the benign lesion cohort. Notably, the benign uptake findings in this study were not restricted to a single pathology but comprised a heterogeneous spectrum of fibro-inflammatory and non-malignant conditions encountered in routine clinical practice, including reactive lymph nodes, inflammatory uptake, postoperative changes, and physiological uptake across multiple anatomical sites. A detailed breakdown is provided in Appendix A, Table A2.

Subgroup analysis based on tumor type demonstrated comparable diagnostic performance of SUVmax across the major cancer groups included in the study. Early and delayed imaging yielded similar AUC values, with only minor and inconsistent variations observed between time points. Specifically, in colorectal cancer, early SUVmax showed an AUC of 0.92 compared with 0.89 on delayed imaging. In pancreatic cancer, delayed imaging demonstrated a slight increase in performance (0.79 vs. 0.74), while in gastric cancer, a modest improvement was also observed (0.87 vs. 0.84). In contrast, appendix cancer showed a small decrease in AUC on delayed imaging (0.72 vs. 0.78). Thyroid cancer demonstrated overall lower diagnostic performance, with a slight increase in delayed imaging (0.59 vs. 0.44) (Table 6).

Overall, the differences between early and delayed SUVmax were small and did not demonstrate a consistent trend favoring either time point. These findings suggest that the limited added value of delayed imaging is consistent across different tumor types and does not appear to be tumor-specific.

## 4. Discussion

In this study, evaluating dual-time-point [^68^Ga]Ga-FAPI PET/CT in a heterogeneous cohort of 123 patients with 620 uptake findings (49.5% malignant, 50.5% benign), early imaging (mean 26 min post-injection) demonstrated diagnostic performance comparable with delayed imaging (mean 65 min), with no clinically meaningful benefit from dual-time-point acquisition. Both malignant and benign uptake findings exhibited significant reductions in SUVmax over time; however, this decline was less pronounced in malignant uptake findings compared with benign uptake findings (%ΔSUVmax −3.6% vs. −7.7%; *p* = 0.0045). TBRmax showed a modest increase over time, reaching statistical significance only in malignant uptake findings, whereas MTV and TLU remained largely unchanged. Receiver operating characteristic analysis confirmed that both early and delayed SUVmax and TBRmax provided only moderate discriminative ability (AUC ~0.65–0.69), while delta parameters demonstrated poor diagnostic performance (AUC approaching 0.5).

The limited temporal variation observed in our cohort suggests that [^68^Ga]Ga-FAPI uptake reaches an early equilibrium phase, with minimal progressive accumulation between approximately 25 and 65 min post-injection. The greater decline in SUVmax observed in benign uptake findings may reflect differences in FAP expression stability, vascular permeability, or tracer washout between malignant and benign tissues. These findings are consistent with prior studies using [^68^Ga]Ga-FAPI-04 and [^18^F]F-FAPI-42, which demonstrated stable uptake and comparable detectability between 30 and 60 min, with early imaging already providing optimal contrast [18,26]. Collectively, these data support the concept that short-interval dual-time-point [^68^Ga]Ga-FAPI imaging provides limited additional biological or diagnostic value beyond a single early acquisition. However, the present study evaluated imaging within the time window routinely used in clinical practice, and our findings should therefore be interpreted within this context. While no meaningful improvement in diagnostic performance was observed with delayed imaging in the evaluated cohort, the possibility that longer standardized delays (e.g., 90–120 min post-injection or beyond) could reveal additional differences in tracer kinetics, lesion-to-background contrast, or diagnostic performance cannot be excluded [27]. Further prospective studies using dedicated late imaging protocols are warranted to address this question.

Despite significantly higher SUVmax and TBRmax values in malignant compared with benign uptake findings at both time points (e.g., early SUVmax 7.9 vs. 4.7; *p* < 0.0001), substantial overlap was observed, resulting in only moderate diagnostic performance. This contrasts with higher AUC values reported in prior single-time-point studies, such as Dabir et al., who demonstrated an AUC of 0.89 for SUVmax [21]. Several factors may explain this discrepancy. First, our cohort included a higher proportion and broader spectrum of benign uptake findings, including post-treatment fibrosis and inflammatory conditions known to exhibit [^68^Ga]Ga-FAPI uptake. Second, a substantial proportion of patients had received prior therapy, which may have induced fibroblast activation and increased nonspecific tracer uptake. Third, the heterogeneity and size of our cohort likely provide a more realistic estimate of performance in routine clinical practice. Importantly, our results demonstrate that delayed imaging does not reduce this overlap, as reflected by the nearly identical AUCs for early and delayed SUVmax and TBR. Delayed imaging demonstrated a slightly higher SUVmax AUC than early imaging (0.687 vs. 0.674); however, the difference was not statistically significant according to DeLong’s test (*p* = 0.18), indicating that delayed acquisition did not provide a statistically significant improvement in diagnostic performance. Furthermore, delta parameters did not improve lesion characterization, indicating that short-interval temporal changes are not diagnostically informative.

Our finding that malignant uptake findings showed higher SUVmax and TBR values, whereas benign findings demonstrated larger MTV and TLU values, may appear counterintuitive but likely reflects differences in the spatial distribution of fibroblast activation. Malignant uptake findings typically exhibit intense, spatially confined FAP expression within tumor-associated stroma, resulting in high SUVmax and TBR values despite relatively small metabolically active volumes. In contrast, benign inflammatory or fibrotic processes often involve a more diffuse pattern of fibroblast activation across larger tissue regions, producing lower uptake intensity but greater volumetric burden. This dissociation highlights that SUVmax and MTV provide complementary information, and reliance solely on intensity-based metrics may underestimate the extent of benign fibro-inflammatory activity.

No consistent tumor-specific pattern favoring delayed imaging was observed; however, these findings should be interpreted cautiously given the limited sample size in several tumor subgroups. SUVmax AUCs were similar between early and delayed imaging in each subgroup, with only minor and inconsistent variations that did not demonstrate a consistent or clinically meaningful trend favoring either time point. For example, in colorectal cancer, early SUVmax showed an AUC of 0.92 compared with 0.89 on delayed imaging, whereas in pancreatic cancer, delayed imaging demonstrated a slight increase (0.79 vs. 0.74), and in appendix cancer, a small decrease (0.78 vs. 0.72). These findings suggest that the temporal behavior of [^68^Ga]Ga-FAPI uptake is not markedly tumor-specific within the evaluated subtypes and that early imaging appears adequate for lesion characterization in this cohort. Although prior treatment may influence FAP expression and tracer kinetics through treatment-related fibrosis and tissue remodeling, no treatment-specific analyses were performed because the primary aim of the study was to evaluate the overall diagnostic value of dual-time-point imaging in a heterogeneous real-world cohort.

The lack of benefit from dual-time-point [^68^Ga]Ga-FAPI imaging contrasts with the established role of delayed imaging in [^18^F]F-FDG PET/CT, where malignant uptake often demonstrates progressive tracer accumulation over time. This difference reflects distinct biological mechanisms. [^68^Ga]Ga-FAPI targets fibroblast activation protein with rapid binding and early plateau of uptake [28,29], whereas [^18^F]F-FDG reflects dynamic glucose metabolism with ongoing intracellular trapping [30]. Therefore, unlike [^18^F]F-FDG, where delayed imaging exploits continued tracer accumulation, [^68^Ga]Ga-FAPI imaging reflects a near steady-state distribution, limiting the utility of time-dependent acquisition strategies.

From a clinical perspective, these findings support the use of early single-time-point [^68^Ga]Ga-FAPI PET/CT (approximately 25–30 min post-injection) as a sufficient protocol for routine imaging. Eliminating delayed acquisition may improve workflow efficiency, reduce patient burden, and increase scanner availability without compromising diagnostic accuracy. However, the moderate diagnostic performance of SUVmax and TBRmax indicates that [^68^Ga]Ga-FAPI PET/CT should not be used in isolation for lesion characterization. Accurate interpretation requires integration with clinical context, anatomical imaging, and, where appropriate, histopathological confirmation. Future approaches to improve specificity may include multiparametric imaging or the development of more tumor-specific tracers.

This study has several limitations. First, its retrospective single-center design may introduce selection bias and limit the generalizability of the findings to other institutions with different patient populations and imaging practices. Second, the lesion-level analysis does not fully account for intra-patient clustering, as multiple uptake findings may originate from the same patient. Although only the most avid lesion per organ was included to reduce overrepresentation, residual within-patient correlation may remain. Third, not all uptake sites were histopathologically confirmed, and a composite reference standard incorporating imaging follow-up, clinical data, and multidisciplinary consensus was used in many cases, potentially introducing classification and verification bias. Fourth, the cohort was heterogeneous with respect to tumor types and prior treatments, and treatment-specific analyses were not performed. In addition, detailed tumor staging and histopathological grading data were not uniformly available, limiting assessment of the influence of tumor biology on [^68^Ga]Ga-FAPI uptake and temporal behavior. Variability in acquisition timing within the predefined early and delayed imaging windows may also have affected quantitative measurements and obscured subtle differences in tracer kinetics. Finally, subgroup analyses in certain tumor categories were limited by relatively small sample sizes.

## 5. Conclusions

In this dual-time-point [^68^Ga]Ga-FAPI PET/CT study, quantitative parameters demonstrated only modest temporal changes, with no statistically significant overall improvement in differentiating malignant from benign uptake findings on delayed imaging. Early imaging provided diagnostic performance comparable to delayed acquisition at the cohort level. Although modest differences were observed in certain tumor-specific subgroup analyses, these findings suggest that a simplified single-time-point protocol at approximately 25–30 min post-injection may be sufficient for most clinical applications. However, given the moderate specificity and substantial overlap between malignant and benign uptake findings, accurate lesion characterization requires integration with anatomical imaging, clinical context, and, when necessary, histopathological confirmation.

## Figures and Tables

**Table 1:** Patient and lesion characteristics.

Characteristic	Value
Total patients	123
Age, years (mean ± SD)	54.4 ± 15.9
Sex	56 males (45.5%) and 67 females (54.5%)
Primary diagnosis, *n* (%)	Colorectal cancer 32 (26.0%); pancreatic cancer 23 (18.7%); gastric cancer 12 (9.8%); appendix cancer 12 (9.8%); thyroid cancer 12 (9.8%); cholangiocarcinoma 9 (7.3%); ovarian cancer 7 (5.7%); carcinoma of unknown origin 4 (3.3%); others 16 (13.0%)
Indication for imaging, *n* (%)	Follow-up 70 (41.9%); therapy response assessment 36 (21.6%); staging 34 (20.4%); restaging 10 (6.0%); suspected recurrence 9 (5.4%); post-surgical evaluation 8 (4.7%).
First imaging time, minutes	25.6 ± 8.4
Second imaging time, minutes	65.2 ± 11.9
Interval between scans, minutes	39.2 ± 8.3

**Table 2:** Comparison of Quantitative Parameters Between Early and Delayed [^68^Ga]Ga-FAPI PET/CT.

Group	Parameter	Early Median [IQR]	Delayed Median [IQR]	Δ Median [IQR]	*p*-Value
Benign	SUVmax	4.70 [3.16–7.70]	4.20 [2.60–7.40]	−0.30 [−1.28–0.20]	0
	MTV	8.91 [4.40–20.01]	10.15 [4.79–20.85]	0.24 [−1.92–3.55]	0.0491
	TLU	25.40 [9.61–76.50]	21.90 [9.28–73.20]	−1.70 [−11.45–4.29]	0.0012
	TBRmax	2.79 [1.77–4.70]	2.86 [1.88–4.59]	0.10 [−0.39–0.51]	0.0517
Malignant	SUVmax	7.90 [4.80–12.60]	7.50 [4.30–12.60]	−0.30 [−1.40–0.90]	0.0194
	MTV	4.00 [1.89–15.20]	4.60 [1.93–15.40]	0.00 [−0.98–1.40]	0.4462
	TLU	17.20 [7.30–74.94]	18.40 [7.40–65.29]	−0.30 [−4.25–3.60]	0.4262
	TBRmax	4.30 [2.63–7.12]	5.00 [2.71–7.83]	0.18 [−0.42–0.98]	0.0001

**Table 3:** Comparison of Percentage Change (%Δ) in Quantitative Parameters Between Malignant and Benign Uptake Findings.

Parameter	Malignant (Median [IQR])	Benign (Median [IQR])	*p*-Value
%ΔSUVmax	−3.6 [−17.4–11.3]	−7.7 [−24.2–4.6]	0.0045
%ΔTBRmax	5.1 [−10.0–22.8]	3.6 [−12.6–23.7]	0.4079
%ΔMTV	0.0 [−22.2–36.4]	4.9 [−22.2–43.7]	0.3616

**Table 4:** Quantitative Parameter Comparison Between Malignant and Benign Uptake Findings.

Parameter	Time Point	Malignant Median (IQR)	Benign Median (IQR)	*p*-Value
SUVmax	Early	7.9 [4.8–12.6]	4.7 [3.2–7.7]	<0.0001
	Delayed	7.5 [4.3–12.6]	4.2 [2.6–7.4]	<0.0001
TBRmax	Early	4.3 [2.6–7.1]	2.8 [1.8–4.7]	<0.0001
	Delayed	5.0 [2.7–7.8]	2.9 [1.9–4.6]	<0.0001
MTV	Early	4.0 [1.9–15.2]	8.9 [4.4–20.0]	<0.0001
	Delayed	4.6 [1.9–15.4]	10.1 [4.8–20.9]	<0.0001
TLU	Early	17.2 [7.3–74.9]	25.4 [9.6–76.5]	0.0465
	Delayed	18.4 [7.4–65.3]	21.9 [9.3–73.2]	0.1191

**Table 5:** Diagnostic Performance of Quantitative Parameters for Differentiating Malignant and Benign Uptake Findings.

Parameter	Time Point	AUC (95% CI)	Cut-Off	Sensitivity	Specificity	PPV	NPV
SUVmax	Early	0.674 (0.631–0.717)	6.94	0.573	0.722	0.669	0.633
	Delayed	0.687 (0.643–0.729)	5.9	0.603	0.684	0.651	0.637
	Δ	0.566 (0.520–0.610)	−13.514	0.72	0.393	0.538	0.589
TBRmax	Early	0.649 (0.605–0.692)	3.667	0.59	0.658	0.627	0.622
	Delayed	0.662 (0.619–0.703)	4.6	0.528	0.754	0.676	0.621
	Δ	0.520 (0.476–0.566)	−19.07	0.905	0.173	0.516	0.651
MTV	Early	0.365 (0.319–0.411)	101.7	0.023	0.984	0.583	0.507
	Delayed	0.362 (0.318–0.406)	144.4	0.02	0.987	0.6	0.507
	Δ	0.480 (0.433–0.527)	−50	0.957	0.071	0.502	0.629
TLU	Early	0.454 (0.409–0.496)	96.16	0.233	0.801	0.534	0.516
	Delayed	0.464 (0.418–0.508)	283.19	0.105	0.929	0.593	0.514
	Δ	0.535 (0.489–0.582)	−30.201	0.83	0.296	0.536	0.639

**Table 6:** SUVmax Diagnostic Performance by Cancer Type.

Cancer Type	*n*	Early SUVmax AUC	Delayed SUVmax AUC
Colorectal cancer	32	0.92	0.893
Pancreatic cancer	23	0.735	0.789
Gastric cancer	12	0.836	0.87
Appendix cancer	12	0.78	0.723
Thyroid cancer	12	0.443	0.585

## Data Availability

The data presented in this study are available on request from the corresponding author. The data are not publicly available due to institutional and privacy restrictions.

## Abbreviations

The following abbreviations are used in this manuscript:

CT	Computed tomography
FAPI	Fibroblast activation protein inhibitor
FDG	Fluorodeoxyglucose
IQR	Interquartile range
MDT	Multidisciplinary team
PET	Positron emission tomography
ROC	Receiver operating characteristic
AUC	Area under the curve
SD	Standard deviation
MTV	Metabolic tumor volume
SUVmax	Maximum standardized uptake value
TBRmax	Maximum tumor-to-background ratio
TLU	Total lesion uptake

## Appendix A

**Table A1:** Distribution of Reference Standards for Lesion Classification.

Reference Standard	Number of Uptake (%)
Histopathology	207 (33.4%)
Composite reference (follow-up + clinical + MDT consensus)	413 (66.6%)

**Table A2:** Distribution of benign findings included in the analysis (*n* = 313).

Benign Finding Category	*n* (%)
Degenerative changes	70 (22.4)
Axillary folds uptake	62 (19.8)
Uterine uptake	42 (13.4)
Liver uptake	29 (9.3)
Pancreatic uptake	26 (8.3)
Benign muscle uptake	24 (7.7)
Splenic uptake	21 (6.7)
Other benign soft-tissue uptake	15 (4.8)
Lung consolidation	12 (3.8)
Benign breast uptake	7 (2.2)
Benign skin uptake	4 (1.3)
Biliary uptake	1 (0.3)
